# Structural characterization and Hirshfeld surface analysis of a Co^II^ complex with imidazo[1,2-*a*]pyridine

**DOI:** 10.1107/S2056989018003857

**Published:** 2018-04-17

**Authors:** Saikat Kumar Seth

**Affiliations:** a Department of Physics, Jadavpur University, Kolkata 700 032, India

**Keywords:** crystal structure, Co^II^ complex with imidazo­pyridine, noncovalent inter­actions, supra­molecular assembly, Hirshfeld surface, fingerprint plot

## Abstract

A new mononuclear tetra­hedral Co^II^ complex, di­chlorido­bis­(imidazo[1,2-*a*]pyridine-κ*N* ^1^)cobalt(II), [CoCl_2_(C_7_H_6_N_2_)_2_], has been synthesized using a bioactive imidazo­pyridine ligand. X-ray crystallography reveals that the solid-state structure of the title complex exhibits both C—H⋯Cl and π–π stacking inter­actions in building supra­molecular assemblies. Indeed, the mol­ecules are linked by C—H⋯Cl inter­actions into a two-dimensional framework, with finite zero-dimensional dimeric units as building blocks, whereas π–π stacking plays a crucial role in building a supra­molecular layered network. An exhaustive investigation of the diverse inter­molecular inter­actions *via* Hirshfeld surface analysis enables contributions to the crystal packing of the title complex to be qu­anti­fied. The fingerprint plots associated with the Hirshfeld surface clearly display each significant inter­action involved in the structure, by qu­anti­fying them in an effective visual manner.

## Abstract 2

The complex [Co*L*_2_Cl_2_] (*L* = imidazo[1,2-*a*]pyridine) exhibits a supra­molecular-layered assembly through π–π stacking inter­actions. The overall inter­molecular inter­actions involved in the structure have been qu­anti­fied and fully described by Hirshfeld surface analysis.

## Chemical context   

In the realm of the synthesis of heterocyclic compounds, imidazo­pyridines have proven to be a most important class of mol­ecules and have attracted significant inter­est because of their promising applications. They are biologically important and have shown a wide variety of pharmacological effects (Adib *et al.*, 2011 ▸): anti-inflammatory (Rupert *et al.*, 2003 ▸), anti­viral (Puerstinger *et al.*, 2007 ▸), anti­ulcer (Kaminski & Doweyko, 1997 ▸), anti­bacterial (Rival *et al.*, 1992 ▸), anti­fungal (Rival *et al.*, 1991 ▸), anti­protozoal (Biftu *et al.*, 2006 ▸; Ismail *et al.* 2008 ▸), anti­herpes (Gudmundsson & Johns, 2007 ▸; Véron *et al.*, 2007 ▸), and for the treatment of hepatitis C (Bravi *et al.*, 2007 ▸), and HIV (Gudmundsson & Boggs, 2007 ▸). These medically relevant compounds exhibit a wide range of activities including anti-herpes, anti­apoptotic, sedative, anxiolytic, anti­convulsant, muscle relaxant, analgesic, anti­tuberculosis and anti­cancer actions (Dymińska, 2015 ▸; Bagdi *et al.*, 2015 ▸). The core structure of imidazo[1,2-*a*]pyridine is present in several drugs, such as zolpidem, alpidem, zolimidine, olprinone, GSK812397, saripidem, and necopidem (Gunja, 2013 ▸; Harrison & Keating, 2005 ▸; Bagdi *et al.*, 2015 ▸). Besides, this heterocyclic scaffold has attracted tremendous attention from the synthetic community due to its prevalence in dyes, ligands for metal catalysts, and electronic materials (Enguehard-Gueiffier & Gueiffier, 2007 ▸; Prostota *et al.*, 2013 ▸; Ke *et al.*, 2013 ▸).

Inspired by the manifold potential applications of imidazo[1,2-*a*]pyridine, we focused our attention on its coord­ination behavior towards metal ions and to the structural features of the resulting complexes. Herein, the crystal and mol­ecular structure of a new Co^II^ complex with imidazo[1,2-*a*]pyridine is described, along with an investigation of the inter­molecular inter­actions *via* Hirshfeld surface analysis.
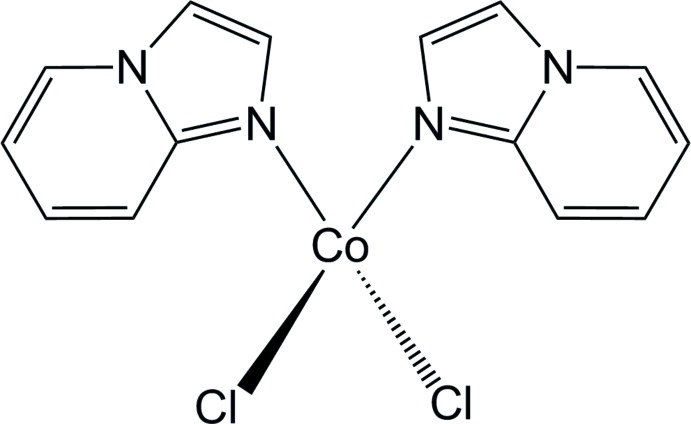


## Structural commentary   

**Figure 1:**
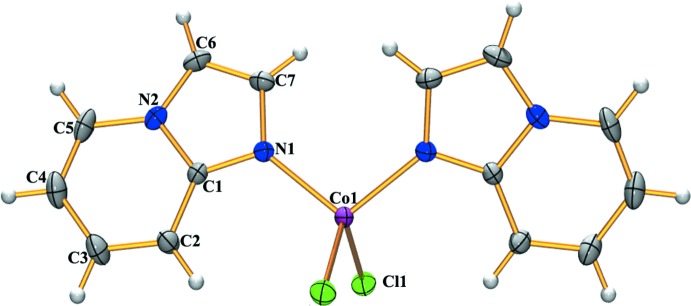
*ORTEP* view with atom-numbering scheme of the title complex with displacement ellipsoids drawn at the 30% probability level. The unlabeled counterpart is generated by the symmetry operation −*x* + 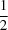, *y*, −*z* + 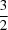.

The mol­ecular structure of the title complex is shown in Fig. 1 ▸. The Co^II^ ion is located on a twofold axis, so that half of the complex is generated by symmetry. The metal center is coord­inated to the nitro­gen atoms of two imidazo­pyridine ligands and to two chlorine ions, and shows a tetra­hedral geometry with angles ranging from 107.70 (5) to 112.44 (5)°. Selected geometric parameters around Co^II^ are reported in Table 1 ▸. The imidazo­pyridine moiety is planar, with a dihedral angle between the rings of 2.47 (3)°. In the imidazo­pyridine moiety, atoms C6 and C4 show the largest deviations in opposite directions [C6: +0.034 (1) and N1: −0.037 (1)] from the least-squares mean plane through the atoms N1/C6/C7/N2/C1–C5.

## Supra­molecular features   

**Figure 2:**
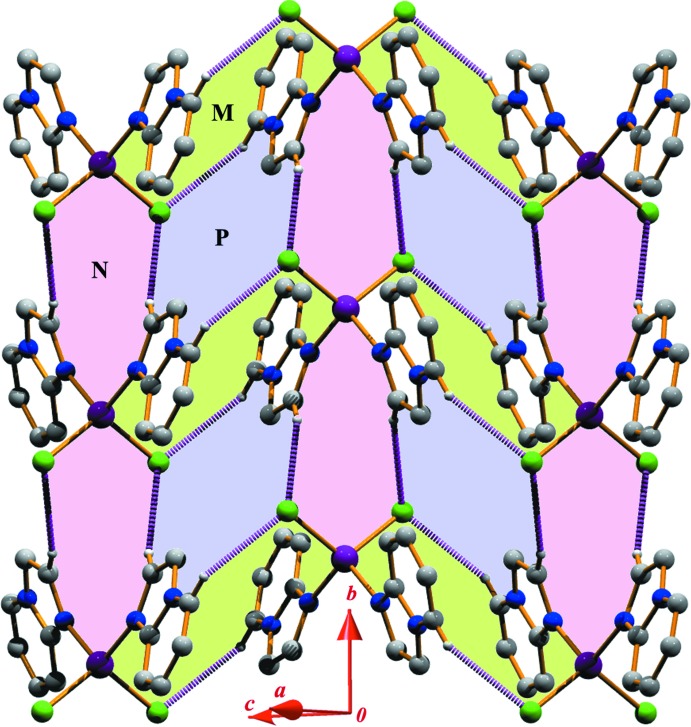
Formation of a two-dimensional supra­molecular network generated through self-complementary C—H⋯Cl inter­actions.

The title structure exhibits inter­molecular C—H⋯Cl and π–π stacking inter­actions; the details are included in Tables 2 ▸ and 3 ▸, respectively. It is convenient to consider the ‘substructures’ generated by each inter­action individually, and then combine these substructures to build up the supra­molecular assembly. The first substructure is formed considering the pyridine ring carbon atom C5 in a general position, which acts as donor to the Cl1 atom at (−*x*, −*y*, 1 − *z*). This C5—H5⋯Cl1 inter­action and its centrosymmetric analogue generate an 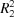
(18) dimeric ring (*M*) centered at (0, 0, 1/2) (Fig. 2 ▸). A second substructure is formed *via* pairs of symmetry-related C7—H7⋯Cl1(*x*, −1 + *y*, *z*) inter­actions, which generate a dimeric 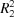
(10) ring (*N)* (Fig. 2 ▸). The propagation of these dimers produces two infinite chains, the first running parallel to the (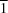
01) plane and the second running parallel to the [010] direction. The inter­connection of the two chains leads to the generation of another tetra­meric 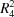
(14) ring motif (*P*). Thus, the two types of 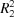
(18) and 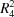
(14) rings are alternately linked into infinite *MPMP*… chains along the [010] direction whereas the 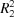
(10) and 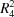
(14) rings are linked alternately into an infinite *NPNP*… chain parallel to the (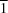
01) plane (Fig. 2 ▸).

**Figure 3:**
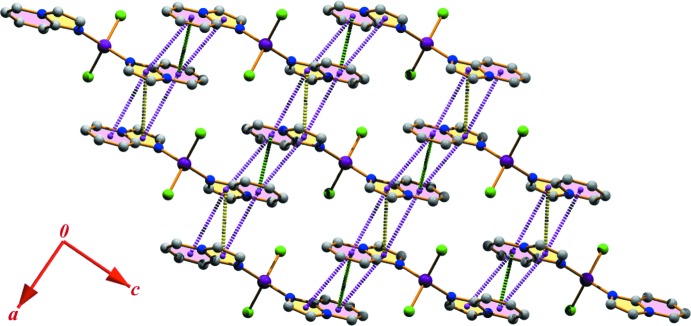
Monomeric units linked through multi π–π stacking inter­actions leading to the formation of a supra­molecular layered assembly. Color codes: the green and yellow dotted lines denote π–π stacking inter­actions between two pyridine rings and two imidazo rings, respectively, whereas π–π stacking inter­actions between pyridine and imidazo rings are represented by pink dotted lines.

Another substructure can be described considering that the mol­ecules, because of their self-complementarity nature, are juxtaposed through π–π stacking inter­actions (Seth *et al.*, 2011*a* ▸, 2013 ▸; Manna *et al.*, 2013 ▸, 2014*a* ▸). The mol­ecular packing of the complex is such that the π–π stacking inter­actions between the pyridine rings, as well as between the imidazo rings, are optimized. The pyridine rings of the mol­ecules at (*x*, *y*, *z*) and (−*x* + 1, −*y*, −*z* + 1) are strictly parallel, with an inter­planar spacing of 3.4671 (9) Å and a ring-centroid separation of 3.5293 (16) Å, corresponding to a ring offset of 0.659 Å. In addition, the imidazo rings at (*x*, *y*, *z*) and (−*x*, −*y*, −*z* + 1) are juxtaposed through face-to-face π-stacking with an inter-centroid separation of 3.6414 (16) Å. Moreover, the imidazo and pyridine rings of the parent mol­ecules are also involved into multi π-stacking inter­actions with each other. In particular, the inter­planar spacing between the imidazo ring in a general position and the pyridine rings at (−*x*, −*y*, −*z* + 1) and (−*x* + 1, −*y*, −*z* + 1) are of 3.5303 (9) and 3.4625 (9) Å, respectively, while the relative ring-centroid separations are 3.9583 (16) and 3.8371 (16) Å. These π–π stacking inter­actions result in a two-dimensional supra­molecular layered assembly parallel to the (010) plane (Fig. 3 ▸).

## Hirshfeld surface analysis   

Mol­ecular Hirshfeld surfaces (Spackman & McKinnon, 2002 ▸) in the crystal structure are constructed considering the electron distribution calculated as the sum of spherical atom electron densities (Spackman & Byrom, 1997 ▸; McKinnon *et al.*, 1998 ▸). The normalized contact distance (*d*_norm_) based on both *d*_e_ and *d*_i_, and the van der Waals (vdw) radii of the atom, given by the equation 
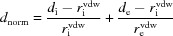
 enable the identification of the regions of particular importance to inter­molecular inter­actions (McKinnon *et al.*, 2007 ▸). The combination of *d*_e_ and *d*_i_ in the form of a two-dimensional fingerprint plot (Rohl *et al.*, 2008 ▸) provides a summary of the inter­molecular contacts in the crystal (Spackman & McKinnon, 2002 ▸). The Hirshfeld surfaces are mapped with *d*_norm_, and the two-dimensional fingerprint plots presented in this work were generated using *CrystalExplorer 3.1* (Wolff *et al.*, 2012 ▸).

**Figure 4:**
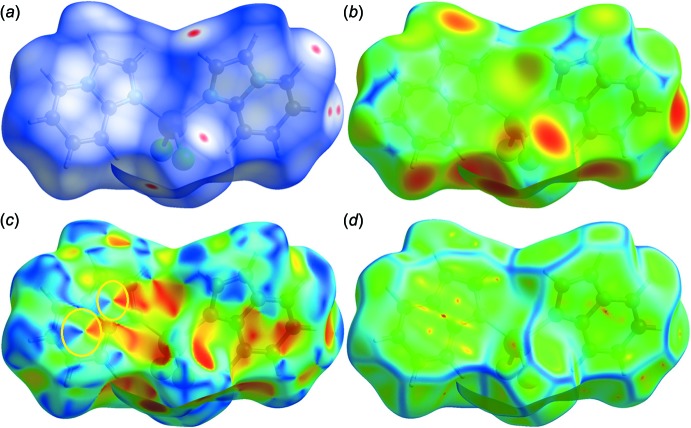
Hirshfeld surfaces of the title complex mapped with (*a*) *d*_norm_, (*b*) *d*_e_, (*c*) shape-index and (*d*) curvedness.

The pattern of the inter­molecular inter­actions of the solid-state structure of the title complex prompted us to explore and qu­antify the contribution of the non-covalent inter­actions in the crystal packing, as well as the importance of the C—H⋯Cl bonding in directing the organization of the extended supra­molecular network (Seth *et al.*, 2011*a* ▸,*b* ▸, Manna *et al.*, 2012 ▸; Seth, 2013 ▸; Mitra *et al.*, 2014 ▸). In this present investigation, the contacts responsible for building the supra­molecular assembly were evaluated with respect to their contribution to the overall stability of the crystal structure. In this context, the Hirshfeld surface analysis (Spackman & McKinnon, 2002 ▸; Seth *et al.*, 2011*a* ▸,*b* ▸,*c* ▸,*d* ▸; Mitra *et al.*, 2013 ▸) of the title complex was performed and the results are illustrated in Fig. 4 ▸. The surfaces represented were mapped over *d*_norm_, *d*_e_, shape-index and curvedness in the ranges −0.0620 to 0.9660 Å, 1.0626 to 2.4714 Å, −1.0000 to 1.0000 Å and −4.0000 to 0.4000 Å, respectively. The information regarding the inter­molecular inter­actions summarized in Tables 2 ▸ and 3 ▸ are visible as spots on the Hirshfeld surfaces (Fig. 4 ▸). For instance, the distinct circular depressions (red spots) on the *d*_norm_ surface (Fig. 4 ▸ *a*) are due to the C—H⋯Cl contacts, whereas other visible spots are due to H⋯H contacts. From the Hirshfeld surfaces, it is also evident that the mol­ecules are related to one another by π–π stacking inter­actions, as can be inferred from inspection of the adjacent red and blue triangles (highlighted by yellow circles) on the shape-index surface (Fig. 4 ▸ *c*). Indeed, the pattern of red and blue triangles in the same region of the shape-index surface is characteristic of π–π stacking inter­actions; the blue triangles represent convex regions resulting from the presence of ring carbon atoms of the mol­ecule inside the surface, while the red triangles represent concave regions caused by carbon atoms of the π-stacked mol­ecule above it. The presence of π–π stacking is also evident in the flat region toward the bottom of both sides of the mol­ecules and is clearly visible on the curvedness surface (Fig. 4 ▸ *d*): the shape of the blue outline on the curvedness surface unambiguously delineates the contacting patches of the mol­ecules. On the *d*_e_ surface, this feature appears as a relatively flat green region where the contact distances are similar (Fig. 4 ▸ *b*).

The inter­molecular inter­actions present in the structure are also visible on the two-dimensional fingerprint plot (Rohl *et al.*, 2008 ▸; Samanta *et al.*, 2014 ▸; Seth, 2014*a* ▸,*b* ▸,*c* ▸), which can be decomposed to qu­antify the individual contributions of each inter­molecular inter­action involved in the structure (Manna *et al.*, 2014*b* ▸). These complementary regions are visible in the fingerprint, where one mol­ecule acts as donor (*d*_e_ > *d*_i_) and the other as an acceptor (*d*_i_ > *d*_e_). Table 4 ▸ contains the percentages of contributions for a variety of contacts in the crystal structure of the title compound.

**Figure 5:**
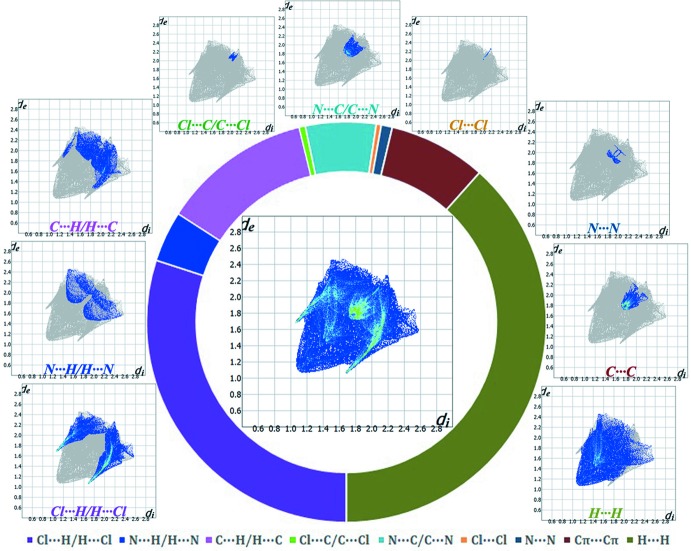
Fingerprint plots: full (middle) and decomposed plots corresponding to all contacts involved in the structure [clockwise: from bottom left to bottom right]. The relative contributions of various inter­molecular contacts to the Hirshfeld surface area of the title structure are displayed by the schematic illustration.

The C—H⋯Cl inter­actions appear as two distinct spikes in the fingerprint plot (Fig. 5 ▸) of the title complex, where Cl⋯H inter­actions have a larger contribution (18.4%) than their H⋯Cl counterparts (11.6%). Thus, the sum of Cl⋯H/H⋯Cl inter­actions comprises 30.0% of the total Hirshfeld surface area of the mol­ecule (Table 4 ▸). The Cl⋯H/H⋯Cl inter­actions represented by the spikes in the bottom right and left region (*d*_e_ + *d*_i_ ≃ 2.77 Å) indicate that the hydrogen atoms from the ligand moiety are in contact with the metal-coord­inated Cl atoms to build the two-dimensional supra­molecular framework. The spoon-like tips in the region (*d*_e_ + *d*_i_ ≃ 3.37 Å) of the fingerprint plot (Fig. 5 ▸) represent a significant N⋯H/H⋯N contribution, covering 4.1% of the total Hirshfeld surface of the mol­ecules. The forceps-like tips in the region (*d*_e_ + *d*_i_ ≃ 3.12 Å) of the fingerprint plot (Fig. 5 ▸) represent the C⋯H/H⋯C contacts where the C⋯H counterpart shows a larger contribution (7.6%) than the H⋯C counterpart (4.5%). Overall, the C⋯H/H⋯C inter­actions account for 12.1% of the total Hirshfeld surface of the mol­ecules (Table 4 ▸), and the carbon atoms of the imidazo­pyridine moiety mainly act as donors in building the mol­ecular assembly. The scattered points in the breakdown of the fingerprint plot show that the π–π stacking inter­actions comprise 7.9% of the total Hirshfeld surface of the mol­ecule (Table 5 ▸) displayed as a region of blue/green color on the diagonal at around *d*_e_ ≃ *d_i_* ≃ 1.743 Å. Another contribution comes from H⋯H contacts (38.4%) represented by the scattered points in the fingerprint plots, and spread up only to *d_i_* = *d*_e_ = 1.092 Å (Fig. 5 ▸).

**Figure 6:**
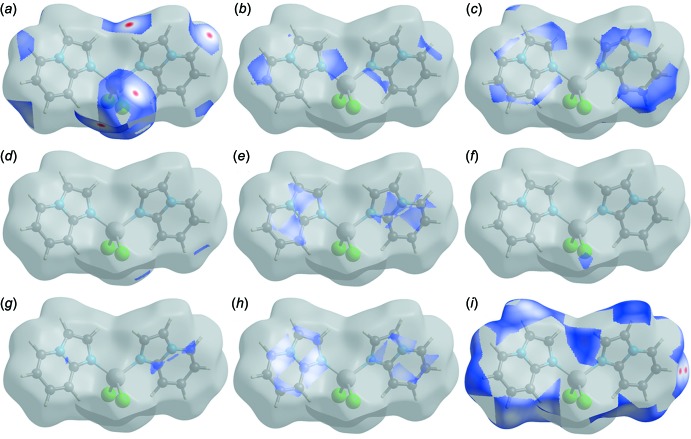
Perspective view of the decomposed *d*_norm_ surfaces of the title structure corresponding to (*a*) Cl⋯H/H⋯Cl; (*b*) N⋯H/H⋯N; (*c*) C⋯H/H⋯C; (*d*) Cl⋯C/C⋯Cl; (*e*) N⋯C/C⋯N; (*f*) Cl⋯Cl; (*g*) N⋯N; (*h*) C⋯C and (*i*) H⋯H contacts.

Finally, the short inter-atomic contacts of the structure (Table 5 ▸) of the type Cl⋯C/ C⋯Cl, N⋯C/ C⋯N, Cl⋯Cl and N⋯N are clearly visible as scattered points in the region *d*_e_ + *d_i_* ≃ 4.07 Å, *d*_e_ + *d*_i_ ≃ 3.58 Å, *d*_e_ + *d*_i_ ≃ 4.11 Å and *d*_e_ + *d_i_* ≃ 3.82 Å of the breakdown fingerprint plots (Fig. 5 ▸). They contribute 0.5%, 5.7%, 0.4% and 0.9%, respectively, to the total Hirshfeld area of the title complex (Table 4 ▸, see Fig. 6 ▸).

The individual inter­molecular inter­actions described above and the qu­anti­tative contributions included in Table 4 ▸ can be also visualized by the different *d*_norm_ surfaces shown in Fig. 6 ▸, confirming that the Hirshfeld surface analysis provides a full understanding of the inter­molecular inter­actions in a facile and immediate way.

## Database survey   

A search in the Cambridge Structural Database (Version 5.38, update May 2017; Groom *et al.*, 2016 ▸) for structures of the general formula [*ML*_2_ *X*_2_], where *M* is any transition metal, *L* is the ligand imidazo[1,2-*a*]pyridine, and *X* any halogen, yielded no results. However, two related complexes exist, with ruthenium and tin, respectively: (i) di­chloro-[2,2′-(pyridine-2,6-di­yl)bis­(imidazo[1,2-*a*]pyridine)]tri­phenyl­phosphine­ruthenium(II) (GULNEI; Li *et al.*, 2015 ▸); (ii) di­bromo-bis(imidazo[1,2-*a*]pyridine)­dimethyl­tin (NODREF; Agrawal *et al.*, 2014 ▸). In both cases, the presence of the halogen atoms is relevant to the stabilization of the crystal structure. In the case of the ruthenium compound, the complex mol­ecules are linked into discrete supra­molecular dimers through pairs of C—H(imidazo)⋯Cl inter­actions. On the other hand, the tin complex forms undulating sheets parallel to the (100) plane by means of C—H(pyridine)⋯Br inter­actions in which both the Br ions and the ligands of one complex act as acceptor and donor, respectively.

## Synthesis and crystallization   

The title complex was prepared by simple hydro­thermal reaction. CoCl_2_·6H_2_O (2.0 mmol, 0.476 g) was dissolved in water (20 ml) yielding a clear pink solution. A hot water–methanol (1:1) solution (20 ml) of imidazo[1,2-*a*]pyridine (1.0 mmol, 0.118 g) was added dropwise to the above solution under continuous stirring. The solution mixture thus obtained was further heated at 343 K for 2 h and then kept for crystallization at room temperature (303 K). The resulting solution was allowed to evaporate slowly at room temperature for several weeks, yielding testable dark-pink crystals, which were collected by filtration, washed with water and dried in air.

## Refinement details   

Crystal data, data collection and structure refinement details are summarized in Table 6 ▸. The hydrogen atoms were located in the difference-Fourier map and refined as riding atoms, with C—H = 0.93 Å and *U*_iso_(H) = 1.2*U*_eq_(C).

## Supplementary Material

Crystal structure: contains datablock(s) I. DOI: 10.1107/S2056989018003857/xi2004sup1.cif

Structure factors: contains datablock(s) I. DOI: 10.1107/S2056989018003857/xi2004Isup2.hkl

CCDC reference: 1583014

Additional supporting information:  crystallographic information; 3D view; checkCIF report

## Figures and Tables

**Table 1:** Selected geometric parameters (Å, °)

Co1—N1	2.0168 (4)	Co1—Cl1	2.2556 (5)
			
N1—Co1—N1^i^	107.70 (5)	N1^i^—Co1—Cl1	112.44 (5)
N1—Co1—Cl1	106.83 (1)	Cl1—Co1—Cl1^i^	110.64 (4)

Symmetry code: (i) 
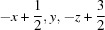
.

**Table 2:** Hydrogen-bond geometry (Å, °)

*D*—H⋯*A*	*D*—H	H⋯*A*	*D*⋯*A*	*D*—H⋯*A*
C5—H5⋯Cl1^ii^	0.93	2.89	3.663 (1)	141
C7—H7⋯Cl1^iii^	0.93	2.88	3.734 (1)	153

Symmetry codes: (ii) 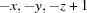; (iii) 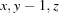.

**Table 3:** Geometrical parameters (Å, °) for π–π stacking (*a*) *Cg*1 and *Cg*2 are the centroids of the (N1/C1/N2/C6/C7) and (N2/C1–C5) rings, respectively; (*b*) centroid–centroid distance between ring *i* and ring *j*; (*c*) vertical distance from ring centroid *i* to ring *j*; (*d*) vertical distance from ring centroid *j* to ring *i*; (*e*) dihedral angle between the first ring mean plane and the second ring mean plane of the partner mol­ecule; (*f*) angle between the centroid of the first ring and the second ring; (*g*) angle between the centroid of the first ring and the normal to the mean plane of the second ring of the partner mol­ecule.

Rings *i*–*j^*a*^*	*Rc^*b*^*	*R*1*v^*c*^*	*R*2*v^*d*^*	α*^*e*^*	β*^*f*^*	γ*^*g*^*	Slippage
*Cg*1⋯*Cg*1^ii^	3.6414 (16)	−3.4980 (8)	−3.4980 (8)	0.0	16.13	16.13	1.012
*Cg*1⋯*Cg*2^ii^	3.9583 (16)	−3.5303 (9)	−3.5035 (9)	2.47	27.73	26.89	–
*Cg*1⋯*Cg*2^iv^	3.8371 (16)	3.4625 (9)	3.4846 (9)	2.47	24.75	25.53	–
*Cg*2⋯*Cg*2^iv^	3.5293 (16)	3.4671 (9)	3.4671 (9)	0.0	10.77	10.77	0.659

Symmetry codes: (ii) −*x*, −*y*, −*z* + 1; (iv) −*x* + 1, −*y*, −*z* + 1.

**Table 4:** Percentage contributions of inter­atomic contacts to the Hirshfeld surface

Contact	% contribution	Contact	% contribution
Cl⋯H/H⋯Cl	30.0	Cl⋯Cl	0.4
N⋯H/H⋯N	4.1	N⋯N	0.9
C⋯H/H⋯C	12.1	C⋯C	7.9
Cl⋯C/C⋯Cl	0.5	H⋯H	38.4
N⋯C/C⋯N	5.7		

**Table 5:** Summary of the short inter­atomic contacts (Å)

Contact	Distance	Contact	Distance
Cl1⋯H7^v^	2.883	C2⋯H6^v^	2.992
Cl1⋯C2^i^	3.613 (2)	C2⋯C5^iv^	3.535 (3)
Cl1⋯H2^i^	2.932	C2⋯H3^vii^	3.021
Cl1⋯H5^ii^	2.893	C4⋯C4^viii^	3.525 (3)
Cl1⋯H3^vi^	3.055	C4⋯H4^viii^	2.834
N1⋯N1^i^	3.257 (2)	C6⋯H2^iii^	3.050
C1⋯C4^iv^	3.482 (3)	H2⋯H3^vii^	2.416
C1⋯C5^iv^	3.516 (3)	H4⋯H4^viii^	2.309
C1⋯C6^ii^	3.518 (3)	H6⋯H2^iii^	2.535

Symmetry codes: (i) −*x* + 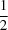, *y*, −*z* + 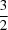; (ii) −*x*, −*y*, −*z* + 1; (iii) *x*, *y* − 1, *z*; (iv) −*x* + 1, −*y*, −*z* + 1; (v) *x*, *y* + 1, *z*; (vi) *x* − 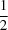, −*y* + 1, *z* + 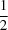; (vii) −*x* + 1, −*y* + 1, −*z* + 1; (viii) −*x* + 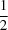, *y*, −*z* + 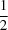;

**Table 6:** Experimental details

Crystal data	
Chemical formula	[CoCl_2_(C_7_H_6_N_2_)_2_]
*M*_r_	366.11
Crystal system, space group	Monoclinic, *P*2/*n*
Temperature (K)	293
*a*, *b*, *c* (Å)	7.712 (2), 6.7898 (18), 14.348 (4)
β (°)	98.533 (5)
*V* (Å^3^)	743.0 (4)
*Z*	2
Radiation type	Mo *K*α
μ (mm^−1^)	1.51
Crystal size (mm)	0.17 × 0.11 × 0.06

Data collection	
Diffractometer	Bruker *SMART* APEXII CCD area-detector
Absorption correction	Multi-scan (*SADABS*; Bruker, 2007 ▸)
*T*_min_, *T*_max_	0.82, 0.92
No. of measured, independent and observed [*I* > 2σ(*I*)] reflections	6663, 1307, 1241
*R*_int_	0.023
(sin θ/λ)_max_ (Å^−1^)	0.595

Refinement	
*R*[*F* ^2^ > 2σ(*F* ^2^)], *wR*(*F* ^2^), *S*	0.024, 0.064, 1.06
No. of reflections	1307
No. of parameters	96
H-atom treatment	H-atom parameters constrained
Δρ_max_, Δρ_min_ (e Å^−3^)	0.18, −0.32

Computer programs: *APEX2* , *SAINT* and *XPREP* (Bruker, 2007 ▸), *SHELXT2014* (Sheldrick, 2015*a* ▸), *SHELXL2018* (Sheldrick, 2015*b* ▸), *ORTEP-3 for Windows* (Farrugia, 2012 ▸), *DIAMOND* (Brandenburg, 2006 ▸), *Mercury* (Macrae *et al.*, 2006 ▸) and *PLATON* (Spek, 2009 ▸).

## supplementary crystallographic information

### Crystal data

**Table:**

[CoCl_2_(C_7_H_6_N_2_)_2_]	*F*(000) = 370
*M**_r_* = 366.11	*D*_x_ = 1.636 Mg m^−^^3^
Monoclinic, *P*2/*n*	Mo *K*α radiation, λ = 0.71073 Å
*a* = 7.712 (2) Å	Cell parameters from 647 reflections
*b* = 6.7898 (18) Å	θ = 1.5–25.0°
*c* = 14.348 (4) Å	µ = 1.51 mm^−^^1^
β = 98.533 (5)°	*T* = 293 K
*V* = 743.0 (4) Å^3^	Block, pink
*Z* = 2	0.17 × 0.11 × 0.06 mm

### Data collection

**Table:**

Bruker SMART APEXII CCD area-detector diffractometer	1307 independent reflections
Radiation source: fine-focus sealed tube	1241 reflections with *I* > 2σ(*I*)
Graphite monochromator	*R*_int_ = 0.023
ω and φ scans	θ_max_ = 25.0°, θ_min_ = 2.8°
Absorption correction: multi-scan (SADABS; Bruker, 2007)	*h* = −8→9
*T*_min_ = 0.82, *T*_max_ = 0.92	*k* = −8→8
6663 measured reflections	*l* = −17→17

### Refinement

**Table:**

Refinement on *F*^2^	Primary atom site location: structure-invariant direct methods
Least-squares matrix: full	Secondary atom site location: difference Fourier map
*R*[*F*^2^ > 2σ(*F*^2^)] = 0.024	Hydrogen site location: inferred from neighbouring sites
*wR*(*F*^2^) = 0.064	H-atom parameters constrained
*S* = 1.06	*w* = 1/[σ^2^(*F*_o_^2^) + (0.0355*P*)^2^ + 0.2499*P*] where *P* = (*F*_o_^2^ + 2*F*_c_^2^)/3
1307 reflections	(Δ/σ)_max_ < 0.001
96 parameters	Δρ_max_ = 0.18 e Å^−^^3^
0 restraints	Δρ_min_ = −0.31 e Å^−^^3^

### Special details

**Table:**

Geometry. All esds (except the esd in the dihedral angle between two l.s. planes) are estimated using the full covariance matrix. The cell esds are taken into account individually in the estimation of esds in distances, angles and torsion angles; correlations between esds in cell parameters are only used when they are defined by crystal symmetry. An approximate (isotropic) treatment of cell esds is used for estimating esds involving l.s. planes.
Refinement. Refinement of F^2^ against ALL reflections. The weighted R-factor wR and goodness of fit S are based on F^2^, conventional R-factors R are based on F, with F set to zero for negative F^2^. The threshold expression of F^2^ > 2sigma(F^2^) is used only for calculating R-factors(gt) etc. and is not relevant to the choice of reflections for refinement. R-factors based on F^2^ are statistically about twice as large as those based on F, and R- factors based on ALL data will be even larger.

### Fractional atomic coordinates and isotropic or equivalent isotropic displacement parameters (Å^2^)

**Table:**

	*x*	*y*	*z*	*U*_iso_*/*U*_eq_	
Co1	0.250000	0.21247 (5)	0.750000	0.03788 (13)	
Cl1	0.00699 (6)	0.40150 (8)	0.73557 (4)	0.05261 (16)	
N1	0.2270 (2)	0.0373 (2)	0.63532 (10)	0.0410 (3)	
N2	0.2373 (2)	−0.0539 (2)	0.48712 (11)	0.0452 (4)	
C2	0.3250 (3)	0.2781 (3)	0.52292 (15)	0.0513 (5)	
H2	0.341071	0.383348	0.564797	0.062*	
C1	0.2664 (2)	0.0958 (3)	0.55145 (12)	0.0386 (4)	
C6	0.1741 (3)	−0.2106 (3)	0.53192 (17)	0.0554 (5)	
H6	0.141218	−0.332623	0.505604	0.066*	
C5	0.2770 (3)	−0.0324 (4)	0.39615 (14)	0.0635 (6)	
H5	0.261300	−0.136802	0.353786	0.076*	
C7	0.1689 (3)	−0.1537 (3)	0.62194 (16)	0.0503 (5)	
H7	0.131285	−0.232355	0.668094	0.060*	
C3	0.3582 (3)	0.2988 (4)	0.43289 (15)	0.0629 (6)	
H3	0.394972	0.419967	0.412794	0.075*	
C4	0.3379 (3)	0.1406 (5)	0.37060 (15)	0.0677 (7)	
H4	0.367003	0.155512	0.310375	0.081*	

### Atomic displacement parameters (Å^2^)

**Table:**

	*U*^11^	*U*^22^	*U*^33^	*U*^12^	*U*^13^	*U*^23^
Co1	0.0447 (2)	0.0343 (2)	0.0365 (2)	0.000	0.01225 (14)	0.000
Cl1	0.0507 (3)	0.0496 (3)	0.0578 (3)	0.0098 (2)	0.0093 (2)	0.0003 (2)
N1	0.0469 (8)	0.0342 (8)	0.0436 (8)	−0.0029 (6)	0.0121 (7)	−0.0024 (6)
N2	0.0415 (8)	0.0476 (9)	0.0451 (9)	0.0027 (7)	0.0022 (7)	−0.0107 (7)
C2	0.0594 (12)	0.0493 (11)	0.0442 (11)	−0.0098 (9)	0.0045 (9)	0.0033 (9)
C1	0.0383 (9)	0.0406 (10)	0.0367 (9)	0.0018 (7)	0.0049 (7)	−0.0037 (7)
C6	0.0561 (12)	0.0403 (11)	0.0677 (14)	−0.0017 (9)	0.0023 (10)	−0.0165 (9)
C5	0.0534 (12)	0.0963 (18)	0.0387 (11)	0.0085 (13)	−0.0003 (9)	−0.0252 (12)
C7	0.0520 (11)	0.0353 (9)	0.0644 (12)	−0.0050 (9)	0.0109 (9)	0.0038 (9)
C3	0.0607 (13)	0.0808 (16)	0.0454 (11)	−0.0161 (12)	0.0019 (10)	0.0154 (11)
C4	0.0540 (13)	0.111 (2)	0.0381 (11)	−0.0105 (14)	0.0054 (9)	0.0056 (13)

### Geometric parameters (Å, º)

**Table:**

Co1—N1	2.0168 (4)	C2—C1	1.400 (3)
Co1—N1^i^	2.0169 (15)	C2—H2	0.9300
Co1—Cl1	2.2556 (5)	C6—C7	1.355 (3)
Co1—Cl1^i^	2.2556 (7)	C6—H6	0.9300
N1—C1	1.344 (2)	C5—C4	1.337 (4)
N1—C7	1.376 (2)	C5—H5	0.9300
N2—C1	1.369 (2)	C7—H7	0.9300
N2—C6	1.370 (3)	C3—C4	1.391 (4)
N2—C5	1.392 (3)	C3—H3	0.9300
C2—C3	1.361 (3)	C4—H4	0.9300
			
N1—Co1—N1^i^	107.70 (5)	N2—C1—C2	119.15 (17)
N1—Co1—Cl1	106.83 (1)	C7—C6—N2	106.79 (17)
N1^i^—Co1—Cl1	112.44 (5)	C7—C6—H6	126.6
N1—Co1—Cl1^i^	112.44 (5)	N2—C6—H6	126.6
N1^i^—Co1—Cl1^i^	106.83 (5)	C4—C5—N2	119.0 (2)
Cl1—Co1—Cl1^i^	110.64 (4)	C4—C5—H5	120.5
C1—N1—C7	105.44 (16)	N2—C5—H5	120.5
C1—N1—Co1	123.48 (12)	C6—C7—N1	110.28 (19)
C7—N1—Co1	131.06 (14)	C6—C7—H7	124.9
C1—N2—C6	107.13 (16)	N1—C7—H7	124.9
C1—N2—C5	121.17 (19)	C2—C3—C4	120.7 (2)
C6—N2—C5	131.65 (19)	C2—C3—H3	119.6
C3—C2—C1	118.9 (2)	C4—C3—H3	119.6
C3—C2—H2	120.5	C5—C4—C3	120.9 (2)
C1—C2—H2	120.5	C5—C4—H4	119.6
N1—C1—N2	110.35 (16)	C3—C4—H4	119.6
N1—C1—C2	130.48 (17)		
			
N1^i^—Co1—N1—C1	−157.31 (17)	C5—N2—C1—C2	4.7 (3)
Cl1^i^—Co1—N1—C1	−39.88 (15)	C3—C2—C1—N1	179.1 (2)
Cl1—Co1—N1—C1	81.68 (14)	C3—C2—C1—N2	−2.6 (3)
N1^i^—Co1—N1—C7	24.35 (15)	C1—N2—C6—C7	−0.9 (2)
Cl1^i^—Co1—N1—C7	141.78 (16)	C5—N2—C6—C7	176.8 (2)
Cl1—Co1—N1—C7	−96.66 (17)	C1—N2—C5—C4	−2.7 (3)
C7—N1—C1—N2	−1.1 (2)	C6—N2—C5—C4	180.0 (2)
Co1—N1—C1—N2	−179.77 (11)	N2—C6—C7—N1	0.2 (2)
C7—N1—C1—C2	177.4 (2)	C1—N1—C7—C6	0.5 (2)
Co1—N1—C1—C2	−1.3 (3)	Co1—N1—C7—C6	179.08 (14)
C6—N2—C1—N1	1.2 (2)	C1—C2—C3—C4	−1.3 (3)
C5—N2—C1—N1	−176.69 (16)	N2—C5—C4—C3	−1.3 (3)
C6—N2—C1—C2	−177.42 (18)	C2—C3—C4—C5	3.4 (4)

Symmetry code: (i) −*x*+1/2, *y*, −*z*+3/2.

### Hydrogen-bond geometry (Å, º)

**Table:**

*D*—H···*A*	*D*—H	H···*A*	*D*···*A*	*D*—H···*A*
C5—H5···Cl1^ii^	0.93	2.89	3.663 (1)	141
C7—H7···Cl1^iii^	0.93	2.88	3.734 (1)	153

Symmetry codes: (ii) −*x*, −*y*, −*z*+1; (iii) *x*, *y*−1, *z*.
